# Comparison of Open and Robot-Assisted Kidney Transplantation in terms of Perioperative and Postoperative Outcomes

**DOI:** 10.1155/2022/2663108

**Published:** 2022-05-06

**Authors:** Serdar Karadag, Mithat Eksi, Osman Ozdemir, Taner Kargi, Ahmet Haciislamoglu, Ismail Evren, Hakan Polat, Dogukan Sokmen, Deniz Noyan Ozlu, Selcuk Sahin, Volkan Tugcu

**Affiliations:** ^**1**^ Department of Urology, University of Health Sciences Bakirkoy Dr. Sadi Konuk Training and Research Hospital, Istanbul, Turkey; ^2^Department of Urology, Bahçelievler Memorial Hospital, Istanbul, Turkey

## Abstract

**Background:**

The gold standard treatment method for end-stage renal disease (ESRD) is renal transplantation (RT). RT can be done with open or minimally invasive surgical methods. We aimed to compare the outcomes between patients who underwent robot-assisted renal transplantation (RART) and open renal transplantation (ORT).

**Methods:**

Data of the patients who underwent ORT or RART in two institutions between June 2015 and February 2020 were retrospectively reviewed. Patients who underwent live donor RT were included, and all donor nephrectomy procedures were performed by the laparoscopic technique. Demographic data, ischemia times, anastomosis times, operation times, and postoperative complications were recorded.

**Results:**

98 patients were included in the ORT group, while 91 patients were included in the RART group. There was a significant difference between the two groups regarding mean patient age. While total ischemia time was 86.9 ± 7 minutes in the RART group, it was calculated as 71.2 ± 3.3 minutes in the ORT group, with a significant difference. The anastomosis time was significantly shorter in the ORT group than in the RART group. The incision length and duration of hospital stay were significantly shorter, visual analogue scores were significantly lower, and estimated blood loss was less in the RART group than in the ORT group.

**Conclusion:**

Both ORT and RART are effective and safe methods for treating ESRD. According to our study, RART is associated with relatively longer ischemia times but lower complication rates and higher patient comfort.

## 1. Introduction

Renal transplantation (RT) is the gold standard treatment method for end-stage renal disease (ESRD) [1]. The surgical technique of RT continuously improved by the increasing experience of the surgeons, since it was initially described in 1956 [2, 3].

In line with these improvements, the use of minimally invasive techniques such as laparoscopic renal transplantation (LRT) and robot-assisted renal transplantation (RART) emerged as alternatives to open renal transplantation (ORT) [4–6]. Hoznek et al. and Menon et al. reported the RART technique, and they noted that this technique led to more favorable outcomes than ORT [5, 6]. Subsequently, the RART technique was adopted by several surgeons, and today is being applied by increasingly more transplant centers [3, 7–9].

This study aimed to compare the operative and postoperative outcome parameters between patients who underwent RART and ORT.

## 2. Materials and Methods

After taking approval from the institutional review board, data of the patients who underwent ORT or RART in two institutions between June 2015 and February 2020 were retrospectively reviewed. All patients gave consent for participation in this study. Patients aged between 18 and 75 who underwent live donor RT were included. Patients who underwent deceased-donor RT, those who had autosomal dominant polycystic kidney disease, and those who underwent simultaneous native nephrectomy were excluded.

Routine preoperative laboratory tests, immunological tests, and urinary ultrasonography were performed in all patients. Also, Doppler ultrasonography was performed to evaluate the iliac vessels of the recipients. Computerized tomography angiogram and renal scintigraphy were performed in all donors for assessing the anatomy of renovasculature and split renal functions.

**Figure 1:**
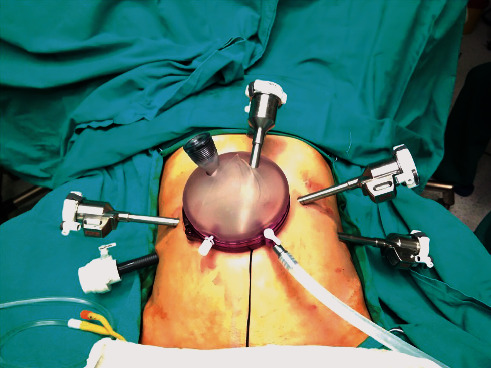
The formation of the ports in the RART technique.

All donor nephrectomy procedures were performed by the laparoscopic technique. The RART procedures were performed using the Da Vinci Xi surgical system (Intuitive Surgical, Sunnyvale, CA, US) by the approach initially described by Menon et al. [5]. The formation of the ports in the RART technique is shown in Figure 1. All RT surgeries were performed by the same transplant surgeon (V.T.) who was significantly experienced in both ORT and RART. The exclusion criteria for patient selection for RART were as follows: severe comorbidities with contraindication for laparoscopic surgery, multiple previous abdominal surgeries, severe iliac vascular calcifications, severely complex vascular anatomy, history of peritoneal dialysis, and autosomal dominant polycystic kidney disease that may preclude laparoscopic surgery.

**Figure 2:**
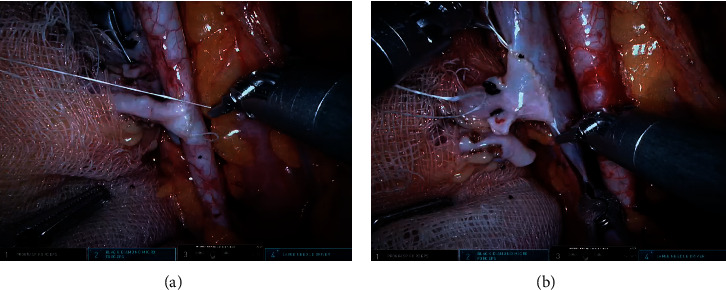
Anastomosis images in RART. Arterial anastomosis (a) and vein anastomosis (b).

**Figure 3:**
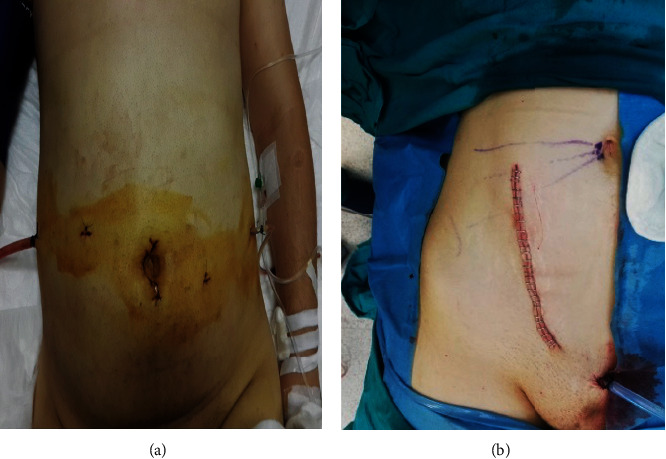
Postoperative incision images. Robot-assisted renal transplantation (a) and open renal transplantation (b).

The ischemia times had been recorded in the patient files. Warm ischemia time was defined as the time interval between clamping the renal artery and immersing the graft in slush ice. Cold ischemia time was defined as the time interval between immersing the graft in slush ice and placement of the graft into the iliac fossa. On the other hand, the time interval between placement of the graft into the iliac fossa and reperfusion of the graft was recorded as the rewarming time. Total ischemia time (TIT) was calculated by taking the sum of warm ischemia, cold ischemia, and rewarming times. Arterial anastomosis time was defined as the time spent during anastomosis of the renal artery to the external iliac artery, and venous anastomosis time was defined as the time spent during anastomosis of the renal vein to the external iliac vein. Anastomosis images in RART are shown in Figure 2. Postoperative incision images are shown in Figure 3. The visual analogue scale (VAS) was used for assessing pain. On this scale, the score of 10 described severe pain, while the score of 0 described being completely painless. Antithymocyte globulin was given for immunosuppression induction to patients with high immunological risk. Prednisone, mycophenolate mofetil, and tacrolimus were given to all patients for maintenance immunosuppression. A routine DJ stent was placed in each patient intraoperatively. Unless contraindicated, Foley's catheter was removed on the third postoperative day and DJ stent was removed in the third week. Delayed graft function (DGF) was defined as a dialysis requirement within the first week after RT. Complications were classified according to the Clavien–Dindo classification [10].

The Statistical Package for Social Sciences (SPSS v22.0, IBM, US) software was used for all statistical analyses. The continuous variables were given as means ± standard deviations. The normal distribution of variables was tested by the Kolmogorov–Smirnov test. The nonnormally distributed variables were presented as medians and interquartile ranges. The Mann–Whitney *U* test was used to compare independent and nonnormally distributed variables, while Student's *t*-test was used for comparing the normally distributed variables. The chi-square test or Fisher's exact test was used to analyze the correlation between categorical variables. The *p* value was considered significant if it was less than 0.05.

## 3. Results

After applying inclusion and exclusion criteria, 98 patients were included in the ORT group, while 91 patients were included in the RART group. Mean patient age was 43.5 ± 11.4 in the ORT and 37.3 ± 10.6 in the RART groups. There was a significant difference between two groups regarding mean patient age (*p* < 0.0001). However, the groups did not differ concerning body mass index (BMI) and gender distribution (*p* > 0.05). There was no significant difference between the two groups in terms of mean operation time (*p*=0.336). While TIT was 86.9 ± 7 minutes in the RART group, it was calculated as 71.2 ± 3.3 minutes ml in the ORT group, with a significant difference (*p* < 0.0001). Comparison of two groups revealed a significant difference regarding anastomosis times; the anastomosis time was significantly shorter in the ORT group than in the RART group (*p* < 0.0001). The incision length, duration of hospital stay, and drain withdrawal time were significantly shorter, VAS scores were significantly lower, and estimated blood loss (EBL) was less in the RART group than the ORT group (*p* < 0.05). The preoperative, operative, and postoperative data of the patients are given in Table 1.

The right kidney was transplanted in 7 cases in the RART group and 9 cases in the ORT group. There were 12 kidneys with multiple renal arteries in the RART group, while 13 kidneys in the ORT group had multiple renal arteries. All transplanted kidneys had a single renal vein and a single ureter.

Thirty-three (33.6%) patients in the ORT group and 36 (39.5%) patients in the RART group underwent preemptive RT, and there was no significant difference between the two groups in this regard (*p* > 0.05). Four (4.08%) patients in the ORT group and 3 (3.29%) patients in the RART group had DGF; the study groups were similar concerning DGF rates (*p* > 0.05). Unless complications developed, routine graft Doppler ultrasonography was not performed in our patients' follow-up. However, no difference was observed in the arterial blood flow of the graft in patients who underwent ultrasound in both groups.

Two patients in the ORT group died in the postoperative period due to sepsis. Among these two patients, one had *Pneumocystis jirovecii* infection and the other had fungal peritonitis due to peritoneal dialysis. Four patients in the ORT group had lymphocele, while 2 had graft thrombosis. Five patients received packed red blood cell (RBC) transfusion in this patient group, and 2 patients had paralytic ileus. One of the patients who had lymphocele was managed by percutaneous drainage, while 3 underwent laparoscopic fenestration. Two patients in the RART group had ileus, and these patients underwent explorative laparotomy. Two patients were temporarily dialyzed. Renal biopsy was performed in these two patients whose creatinine did not return to normal in the first postoperative week, and the biopsies resulted as acute tubular necrosis. Normal creatinine level was achieved in both patients within one month at the latest. There was no risk factor for type and degree of organ matching in both patients, and there was no history of specific intraoperative complications. In addition, one patient received a packed red blood cell transfusion. None of the patients in the RART group had lymphocele. The postoperative complications encountered in the entire cohort are given in Table 2.

## 4. Discussion

Renal transplantation led to a significant decline in ESRD-related mortality [2, 11]. Development of the surgical technique, improvement in patient care, and immunosuppression regimes significantly increased graft survival in the last decades [1]. Because it significantly increased the patients' quality of life and comfort, minimally invasive RT techniques gained popularity among transplant surgeons [11]. However, since laparoscopic RT was described by Modi et al., it did not become popular because it requires advanced laparoscopic surgical skills [4]. The RART technique was initially described by Hoznek et al. [6], and it was subsequently modified by and Menon et al. [5] and Oberholzer et al. [8]. This technique was later adopted and used by several RT centers [9, 12–14].

In our study, analysis of the operative data revealed that surgical times were similar between the two groups. However, the cold ischemia time, rewarming time, and total ischemia time were significantly longer in the RART group than in the ORT group (*p* < 0.0001). Similarly, arterial, venous, and ureterovesical anastomosis times were significantly longer in the RART group than in the ORT group. In line with this, Pein et al. reported that ischemia times were significantly longer in RART than in ORT [15]. However, the authors stated that the duration of surgery (i.e., surgical time) was significantly longer in RART than in ORT. This finding is inconsistent with ours. Of course, since robotic surgery shows an important learning curve, we believe that parameters such as operation and anastomosis time will shorten as experience increases.

In a review analyzing several robotic surgery techniques, the authors concluded that robot-assisted surgeries were associated with significantly less intraoperative blood loss and shorter duration of hospital stay than the other surgical modalities [16]. It was reported that drain removal time and duration of hospital stay were significantly shorter in RART than in ORT [3, 15]. Our study determined that RART was associated with significantly less intraoperative bleeding, shorter drain removal time, and duration of hospital stay than ORT (*p* < 0.05). The postoperative VAS scores were also in favor of RART (*p* < 0.0001).

One of the essential advantages of robotic surgery is relatively low complication rates [15, 16]. In our study, complications were seen in 9 (9.89%) patients in the RART group and in 20 (20.4%) patients in the ORT group. Classification of the complications as per Clavien–Dindo grading revealed that 4 of the 9 patients in the RART group had Clavien–Dindo grade 1 complications. While, there was only 1 patient with wound infection in the RART group and 5 patients in the ORT group. Also, it was observed that the incision length was significantly shorter in the RART group than in the ORT group. These findings are in line with those reported in the literature [17].

One of the most important postoperative complications of RT is lymphocele [18, 19]. Researchers analyzing the RART series reported that lymphocele was significantly less frequent in patients who underwent RART than those who underwent ORT [18, 19]. In our study, there were 4 (4.08%) patients with lymphocele in the ORT group, while none of the patients in the RART group had lymphocele.

Our series represents the most extensive single-surgeon series comparing the patients who underwent ORT and RART concerning operative and postoperative data to the best of our knowledge. However, its retrospective design can be considered as a limitation of this study.

## 5. Conclusion

Both ORT and RART are effective and safe methods for treating ESRD. On the other hand, our study showed that RART is associated with lower complication rates and higher patient comfort, but relatively longer ischemia times, which has the potential to affect graft survival in the long term. Further studies are needed in this respect.

## Figures and Tables

**Table 1:** Demographic, preoperative, perioperative, and postoperative data of all patients and comparison of the groups.

Parameters (mean ± SD)	All	ORT	RART	*P*
Age (year)	40.5 ± 11.4	43.5 ± 11.4	37.3 ± 10.6	**<0.0001**
BMI (kg/m^2^)	24.2 ± 2.7	24.4 ± 2.1	23.9 ± 3.3	0.215
Preoperative				
Hemoglobin (g/dL)	9.9 ± 2	10.3 ± 1.75	9.4 ± 2.2	**0.002**
Creatinine (mg/dL)	6.8 ± 2.5	6.8 ± 1.9	6.7 ± 2.9	0.739
eGFR (mL/min/1.7)	10.8 ± 3.9	10.7 ± 3.3	10.9 ± 4.4	0.707
Operation time (min)	243.8 ± 47.3	240.6 ± 54.4	247.2 ± 38.2	0.336
TIT (min)	78.8 ± 9.5	71.2 ± 3.3	86.9 ± 7	**<0.0001**
Warm ischemia time (min)	1.7 ± 0.2	1.7 ± 0.2	1.7 ± 0.1	**0.005**
Cold ischemia time (min)	34.6 ± 3.1	32.6 ± 2.1	36.8 ± 2.4	**<0.0001**
Rewarming time (min)	42.3 ± 7.3	36.8 ± 2.6	48.3 ± 6.1	**<0.0001**
Arterial anastomosis (min)	15.8 ± 1.8	14.5 ± 1.1	17.3 ± 1.2	**<0.000**
Venous anastomosis (min)	17.9 ± 2.7	16.2 ± 2.1	19.6 ± 2.1	**<0.0001**
UV anastomosis (min)	16.9 ± 3.6	14.8 ± 2.7	19.1 ± 2.1	**<0.0001**
EBL (ml)	191 ± 51.9	211.6 ± 27.3	168.8 ± 62.2	**<0.0001^*∗*^**
Incision length (cm)	8.2 ± 3	10.9 ± 1.3	5.2 ± 0.7	**<0.0001**
Postoperative 1st day				
Hemoglobin (g/dL)	9.4 ± 1.3	9.1 ± 0.9	9.7 ± 1.6	<0.003
Creatinine (mg/dL)	3.8 ± 1.4	3.8 ± 0.8	3.9 ± 1.8	0.644
eGFR (mL/min/1.7)	22 (21)″	24 (12.2)″	18 (21)″	0.550^*∗*^
Length of stay (day)	9 (4.5)″	10 (8)″	10 (3)″	**0.023^*∗*^**
Drain withdrawal (day)	5 ± 1.8	6.5 ± 1.3	3.4 ± 0.6	**<0.0001**
VAS				
Postoperative				
12th hour	6.4 ± 1.1	7 ± 0.9	5.8 ± 1	**<0.0001**
24th hour	5.3 ± 1.3	6.1 ± 0.8	4.5 ± 1.1	**<0.0001**
36th hour	4.3 ± 1.3	4.9 ± 1.1	3.7 ± 1.2	**<0.0001**
48th hour	3.4 ± 1.4	3.9 ± 1.2	2.7 ± 1.2	**<0.0001**

^*∗*^Mann–Whitney *U* test. ″Presented as median (interquartile range). ORT, open renal transplantation; RART, robot-assisted renal transplantation; BMI, body mass index; eGFR, mean glomerular filtration rate; TIT, total ischemia time; UV, ureterovesical; EBL, estimated blood loss; VAS, visual analogue scale.

**Table 2:** Complications according to the Clavien–Dindo classification.

Complications^*∗*^	ORT	RART
Grade I	7: 5 wound infections and 2 paralytic ileus	4: 2 wound infections and 2 paralytic ileus
Grade II	6: 1 orchitis and 5 ERT	1: ERT
Grade IIIa	3: 1 lymphocele and 2 graft thrombosis	—
Grade IIIb	3: lymphocele (laparoscopic fenestration)	2: exploratory laparotomy
Grade IVa	1: sepsis	2: temporary dialysis
Grade V	2: death (sepsis)	—

^*∗*^According to Clavien–Dindo classification. ERT, erythrocyte replacement therapy.

## Data Availability

The data used to support this study are under the control of the government and are official documents and not allowed for public use.
